# Toxic Plants and Their Impact on Livestock Health and Economic Losses: A Comprehensive Review

**DOI:** 10.1155/jt/9857933

**Published:** 2024-12-18

**Authors:** Tagesu Abdisa, Tegegn Dilbato Dinbiso

**Affiliations:** ^1^Chelia District Agricultural and Land Office, Animal Health Protection Team, Chelia District, West Shewa, Oromia, Ethiopia; ^2^Ambo University, Guder Mamo Mezemir Campus, Department of Veterinary Science, West Shewa Zone, Oromia, Ethiopia

**Keywords:** economic loss, health, impacts, livestock, toxic plants

## Abstract

Plants are important components in sustaining the life of humans and animals, balancing ecosystems, providing animal feed and edible food for human consumption, and serving as sources of traditional and modern medicine. However, plants can be harmful to both animals and humans when ingested, leading to poisoning regardless of the quantity consumed. This presents significant risks to livestock health and can impede economic growth. In several developing countries, including Ethiopia, traditional communities have depended on medicinal plants for treating livestock and human diseases. The incidences of livestock poisoning from medicinal and poisonous plants are due to the misuse and lack of dosage standardization. Therefore, this paper aimed to review toxic plants and their effects on livestock health and associated economic losses. Toxic plants contain secondary metabolites that serve as a defense mechanism against predators. The most common secondary metabolites of toxic plants that affect livestock health and the economy include alkaloids (Asteraceae, Convolvulaceae, Lamiaceae, Fabaceae, and Boraginaceae), cyanides (*Sorghum* spp. and grass spp.), nitrates (*Pennisetum purpureum* roots*, Amaranthus,* nightshades*, Solanum* spp. *Chenopodium* spp., and weed spp.), oxalates (Poaecea, Amaranthaceae, and Polygonaceae), and glycosides (*Pteridium aquiline*). The most common effects of toxic plants on livestock health include teratogenic and abortifacient (Locoweeds, Lupines, Poison Hemlock, and Veratrum), hepatoxicity (*Crotalaria, Lantana camara, Xanthium,* and *Senecio*), photosensitization (*L. camara, Alternanthera philoxeroides, Brachiaria brizantha,* and *Heracleum sphondylium*), and impairing respiratory and circulatory systems (nitrite and cyanide toxic). Toxic plants lead to substantial economic losses, both direct and indirect. Direct losses stem from livestock deaths, abortions, decreased milk quality, and reduced skin and hide production, while indirect losses are associated with the costs of treatment and management of affected animals. Overall, toxic plants negatively impact livestock health and production, resulting in significant economic repercussions. Therefore, it is crucial to prioritize the identification of the most prevalent toxic plants, isolate secondary metabolites, conduct toxicity tests, standardize dosages, and develop effective strategies for managing both the toxic plants and their associated toxicity.

## 1. Introduction

Plants play a crucial role in sustaining life on Earth, serving as fundamental components of ecosystems. They contribute to the balance of natural systems by producing oxygen through photosynthesis, which is essential for the survival of both humans and animals. They are also vital for agriculture, supplying animal feed and edible food for human consumption, thereby underpinning food security worldwide [1, 2]. Moreover, plants are a significant source of traditional and modern medicine. Many pharmaceuticals are derived from plant compounds, highlighting their importance in healthcare. Traditional practices often utilize various plant species for their healing properties, contributing to cultural heritage and community well-being. Thus, preserving plant diversity is essential for ecological balance, food security, and health advancements. However, plants can be harmful to both animals and humans when ingested, leading to poisoning regardless of the quantity consumed [3, 4].

Toxic plants have harmful chemicals that poisonous to livestock, leading to a loss of economic production [5]. The study of poisons and the pursuit of effective antidotes began in ancient Greece to eliminate opponents. In ancient times, scientists focused on the knowledge of poisons and poisoning incidents. Before 1550 BC, the deadly chemical was extensively researched all over the world, particularly in Egypt and Greece. Aconitine, opium, cyanogenic glycosides, and alkaloid isolates were among more than 900 prescription medications found in the earliest pharmacopeia of ancient Egyptians [6–8]. Then afterward, Paracelsus, the founder of toxicology, concluded that the primary hazardous component of poisons is their chemical action on organisms and postulated that a single dose of substance determines its toxicity [9–11].

The problem of toxic plants is greater in countries with higher plant biodiversity [5]. According to Dinbiso et al. (2022), estimated floras of 6500–7000 species of higher plants are medically important, and out of these medicinal plants, 12% are endemic to Ethiopia. Ethiopia had a long history of producing poisonous substances from various plant species. The humans collected the poisons and used them for hunting, wars, murder, abortion, and euthanasia. In addition, the neighborhood practiced traditional ethnoveterinary methods to treat and prevent illnesses in both cattle and people [12, 13]. Moreover, medicinal plants have been screened for medicinal purposes against microorganisms at the laboratory level. An experimental investigation screened secondary metabolites in medicinal plants that have medicinal value based on their dosage and route of administration [14].

Currently, assessments of the most common medicinal plants used in the treatment of livestock diseases are being carried out in various districts throughout Ethiopia [14–16]. However, the community employed medicinal plants for therapeutic purposes without considering toxicity. Most substances act as poisonous defense chemicals used to have medicinal properties at lower concentrations. Those include alkaloids, cardiac glycosides, phenols, esters, lectins, and cyanogenic glycosides [17]. Some medicinal plants are intrinsically toxic by their constituents and can cause adverse reactions if inappropriately used [18]. Research has shown that medicinal plants have toxicity against the health of livestock and humans when unregulated and used at a dose-dependent risk of toxicity [19]. Moreover, the toxicity of medicinal plants has not historically been taken into account when treating livestock diseases [20, 21]. Thus, livestock animals have suffered from the toxicity of medicinal and toxic plants. Livestock are exposed to toxic plants when they have accidental ingestion of toxic plants with grass as well as during a shortage of feed [22]. This is the fact that a shortage of feed may occur during the drought when toxic plants remain attractive to hungry livestock. Animals with nutritional deficiencies may be less able to detoxify plant toxins and suffer from their toxicity [23, 24].

The epidemiological implications of toxic plants that cause toxicity in livestock can be based on factors including plant growth stage, toxic plant dose consumed, worm or insect attachment on plants, time of day, rainy season, animal age, species, and body condition [25, 26]. Toxic plants have a significant influence on livestock health by disrupting the function of their bodily systems [15, 27]. Toxic plants cause significant production losses that affect the economies of the countries [28]. Therefore, the objective of this paper was to review the toxic plants and their impact on livestock health and economic losses.

## 2. Literature Review

### 2.1. Toxic Plant and Its Secondary Metabolites

Toxic plants are those that can harm animals and people when ingested in excessive or small dosages and lead to poisoning [29]. The history of poisoning is one of the most important stories in human history, where curiosity and genius, scientific discoveries, and empirical knowledge interact with intrigues and crimes. Toxic compounds have probably existed since the beginning of time. In the Middle Ages, Paracelsus believed that only the dose determines that a thing is not a poison and that toxic quantities of the substance are indistinguishable up to a single parameter dose [11]. In the ancient era, toxic plants were used for euthanasia, abortion, hunting, and suicide purposes. Hunters employ toxic plants that affect the central nervous system as arrow poisons in both hunting and combat. For example, *Conium maculatum* poisons were used for suicide, abortion, and murder in prehistoric times. Coniine, aconitine, atropine, and cardiac glycosides are found in this deadly plant. According to studies, cytotoxic alkaloids and triterpenes, which are used as abortifacient, were abundant in the extracts from *Petroselinum crispum* and *Bryonia dioica* [14, 17].

Plants are unable to flee by movement from the animals and have developed defense mechanisms to prevent harm. These are both physical methods such as thorns and sticky outpourings from wounds and chemical defense methods. The chemical defenses have evolved against invertebrate animals and vertebrate animals. Plants produce toxic chemicals for many reasons, including defense against herbivores and microbial infections [26]. Plants naturally contain a significant variety of phytochemical components that support their development and defensive mechanisms. They are divided into primary and secondary metabolites by their function in the metabolic process [30]. The secondary metabolites are formed from primary metabolites by biosynthetic modifications (methylation, glycosylation, and hydroxylation) that are used for defensive mechanisms rather than growth and development [31].

The nature of the toxic secondary metabolites varies concerning the place of origin and surrounding environmental conditions. The secondary metabolites of plants include tannins, phenolics, alkaloids, phytohemagglutinins, terpenes, cyanogenic glycosides, oxalates, protease inhibitors, lectins, nonprotein amino acids, and saponins [27, 32, 33]. Toxic plants are classified under different families. Among the families, the most common toxic plants are *Fabaceae, Phytolaccaceae, Rutaceae, Pinaceae, Apiaceae, Solanaceae, Asteraceae, Ranunculaceae, Polypodiaceae, Urticaceae, Euphorbiaceae,* and *Polygonaceae* [34]. The most common secondary metabolites of toxic plants that poisonous to livestock include alkaloids (*Asteraceae Convolvulaceae*, *Lamiaceae*, *Fabaceae*, *Boraginaceae*), cyanides (*Sorghum* spp., and grass spp.), nitrates (*Pennisetum purpureum* roots, *Amaranthus*, nightshades (*Solanum* spp.), and *Chenopodium* spp., weeds spp.), oxalates (*Poaecea*, *Amaranthaceae*, and *Polygonaceae*), and glycosides (*Pteridium aquiline*) [32–35], indicated in Table 1.

Plant factors (palatability, stage of plant growth, and plant parts), animal factors (age and breed), nutritional status (hunger and nutritional deficiency), and environmental factors (season, distribution, weather conditions, droughts, and soil type) are the most common predisposing factors that measure the occurrence of toxic plant effects and how livestock exposure to poisoning occurs [26]. The toxic plant that poisons one animal species may not be poisoning other species. Livestock can detoxify some toxic compounds from toxic plants. The effectiveness of detoxification differs depending on the animal species [56]. The microflora and microsomal enzymes of ruminants help in the detoxification of toxin compounds [57]. Toxic plants have distressing effects on the health status of livestock as well as diminish the economic growth of livestock production. Therefore, the most common hazardous effects of toxic plants that impair the livestock's body functional system are categorized as hepatotoxicity, neurotoxicity, nephrotoxicity, GIT system disturbance, effects on the reproductive system, and destruction of skin tissues [27, 28, 57].

### 2.2. Effect of Toxic Plants on Livestock Health

#### 2.2.1. Hepatotoxicity Effect

Toxic plants damage the liver, as it is the first organ exposed to the intestinal portal blood and has many mixed-function oxidases with potential bioactive xenotoxins. Hepatocytes bioactivate toxins, causing damage to different parenchymal sections of the hepatic lobule. Therefore, plant toxins are quickly bioactivated and become potent alkylating agents that denature hepatic proteins and nucleic acids in the first hepatocytes they encounter [58]. Hepatotoxic plants are those that have pyrrolidine alkaloids (PAs), which cause potent liver toxins. Most are unpalatable becoming a problem for livestock, when alternative forages are unavailable and included in hay. PAs induce hepatocyte necrosis that progresses to liver failure and are potent carcinogens at low levels below those causing hepatic necrosis [59, 60]. The most common toxic plants that cause toxicity in the liver belong to the families of Asteraceae (Senecio and Eupatorium), Convolvulaceae, Lamiaceae, Fabaceae (Crotalaria), and Boraginaceae [59, 61].

##### 2.2.1.1. Crotalaria

Crotalaria poisoning in livestock is caused by the ingestion of pyrrolizidine alkaloid-containing plants, resulting in severe liver damage. It is a plant genus belonging to the Fabaceae family and is mainly found in tropical and subtropical regions [62, 63]. All species of livestock are susceptible to this toxic plant. The ingestion of Crotalaria causes Missouri bottom disease, which causes animals to become slow, emaciated, weak, and progressive liver degeneration [59]. Crotalaria contains PAs that are potent hepatotoxic compounds with cytotoxic, genotoxic, and oncogenic effects. Moreover, PAs are bioactivated by the cytochrome P450 (CYP) system forming the toxic derivative pyrrole, which promptly binds to cellular macromolecules (proteins, DNA, and RNA) to form adducts, resulting in cellular degeneration and necrosis [64, 65]. The most clinical signs with concern of damaged liver include jaundice, apathy, a hypotonic tongue, circling, anorexia, frequent yawning, ataxia, weight loss, aimless wandering, and violent behavior [66, 67]. Crotalaria poisoning can be primarily treated with supportive therapy focusing on alleviating clinical signs and preventing further liver damage. Prevention strategies include the identification and removal of Crotalaria plants, weed control, pasture rotation, hay testing, and education of livestock owners (68).

##### 2.2.1.2. *Lantana camara*

It is a common invasive shrub of the Verbenaceae family, which poses a significant threat to livestock due to its hepatotoxic properties. The phytochemistry of *L. camara* is complex as it contains a wide variety of chemical substances, including triterpenes, mono- and sesquiterpenes, iridoid and phenylethanoid glycosides, naphthoquinones, and flavonoids. The hepatotoxic action of *L. camara* has been attributed to two pentacyclic triterpenes known as lantadene A and B [68]. The ingestion of plant foliage by grazing animals causes hepatotoxicity and is an important cause of livestock morbidity and mortality in Lantana-infested regions [40]. The leaves and immature berries are more toxic to livestock. Lantana toxins damage hepatocytes, leading to cholestasis and hepatocyte necrosis. Clinical signs include anorexia, weight loss, jaundice, depression, and reduced milk production. The drastic effects of Lantana poisoning include cholestasis and hepatotoxicity due to the continuous absorption of toxins from the rumen. As a result, ruminal stasis and anorexia develop and other related consequences of cholestasis are jaundice and photosensitization [39]. Treatment involves removing the affected cattle from Lantana-infected areas, providing supportive therapy, administering liver protectants, treating photosensitization, and drenching activated charcoal. To prevent Lantana poisoning, it is essential to control its spread using methods such as mechanical removal and biological control agents, while also closely monitoring livestock for signs of poisoning [5, 40].

*Xanthium* and *Senecio* spp. contain alkaloids that cause hepatotoxicity. These alkaloids have antimitotic effects on hepatocytes that prevent cell division but not DNA synthesis, resulting in nuclear and cytoplasmic enlargement and formation of melanocytes. The main cytologic and histologic abnormality in PA toxicosis is related to chronic bovine liver disease [69, 70].

#### 2.2.2. Teratogenic and Abortifacient Effect

Toxins of toxic plants can efficiently cross the placenta and induce congenital malformations in a fetus at critical periods during gestation, which are called teratogenic effects. In addition, the potential effects of indiscriminate use of medicinal plants are teratogenic, embryotoxic, and abortifacient effects [69, 71]. The most common teratogenic toxic plants include *locoweeds*, *lupines*, *poison hemlock*, and *veratrum*. These toxic plants have different secondary metabolites (toxic compounds) that cause reproductive problems in livestock production [28]. Livestock animals are poisoned by consuming feeds that are contaminated by toxic plants, and the poisoned livestock develop compromised reproductive functions such as infertility, abortion, and fetal deformities [72].

##### 2.2.2.1. Locoweeds

They are biennial and perennial flowering plants that are classified under the Fabaceae family. Locoweeds are found worldwide in mountains, foothills, plains, and semiarid desert regions. Locoweeds contain secondary metabolite compounds such as swainsonine and indolizidine alkaloids [41, 73]. The highest concentrations of swainsonine are found in the flowers and seeds of locoweeds. These plants are palatable, and later, livestock become poisoned when consumed [54]. Ingestion of locoweed rapidly leads to various detrimental effects in pregnant animals. These include the development of cystic ovaries with abnormal estrus cycles, fetotoxic effects causing placental resorption and abortion, fetal myocardial dysfunction with placental damage, and the firmation of excess amniotic fluid (hydramnios). Additionally, locoweed exposure can result in birth defects in offspring, such as malformations of the limbs (phocomelia) [72]. The toxicity of locoweed can be managed by controlling the weeds. The treatment is so difficult in pregnant animals; therefore, avoiding livestock from the consumption of this toxic plant is the best option [28, 73, 74].

##### 2.2.2.2. *Poison hemlock*

It is a biennial herbaceous plant from the Apiaceae family and thrives in moist environments like roadside ditches, and damp waste areas. Its identification is aided by irregular purple spots along the main stem, and a single, carrot-like taproot distinguishes the plant [48]. The plant contains several piperidine alkaloids, notably coniine and coniceine, which are responsible for its toxicity and teratogenic effects [41]. Both the leaves (in spring) and fruits (in fall) pose a significant danger to livestock. When ingested, even in small amounts, poison hemlock can cause severe reproductive issues in livestock, including teratogenic effects such as skeletal birth defects and cleft palate, as well as abortion (28, 50).

##### 2.2.2.3. *Lupines*

It is classified under the *Lupinus* genus and is considered a highly nutritious legume and an important component of livestock and wildlife nutrition. It is expanded in disturbed areas and is common in ranges and pastures that are overgrazed or drought-stressed. Although many lupines appear similar and may even be classified as the same species, there are large population differences with unique alkaloids and concentrations that may produce birth defects. Mostly, cattle suffer more from this teratogenic effect of lupines when the female livestock graze toxic lupines during days mid- and last trimester [42]. The teratogenic effect of this toxic plant is due to the alkaloid-induced reduction in fetal movement during the critical stage of gestation. The most common effect of lupines in cattle teratogenesis is called crooked calf disease. Crooked calf disease is a congenital condition in calves that are born with skeletal contracture-type malformations and cleft palate. It also produces appendicular contractures that most often involve the front legs and include permanent contractures, rotational defects, and massive joint deformation, ankyloses, and bony deformation [41, 75].

##### 2.2.2.4. Veratrum (False Hellebore)

This toxic herb poses a serious threat to sheep, causing poisoning. It blooms during July and August, setting seed in late August and early September. Veratrum remains toxic throughout its life cycle until killed by freezing, with toxicity decreasing as the plant matures [76]. Sheep and goats readily consume the entire plant, and cattle may also eat it if other feed is scarce. The root is 5–10 times more toxic than the leaves and stems. Cyclopamine and steroidal alkaloids are the toxins present in veratrum. When a pregnant sheep consumes veratrum on the 14th day after breeding (early gestation), the fetus may develop *cyclopia* (single eye) and other facial deformities, known as monkey-faced lambs [41, 76].

#### 2.2.3. Cyanogenic Toxicity

Cyanide, a naturally occurring substance in the plant kingdom, is primarily found in the form of cyanogenic glucosides (cyanogens). These cyanogens are relatively concentrated in grasses, pulses, root crops, and fruit kernels, often contributing to their bitter taste [77]. Common cyanogenic plants include those containing amygdalin (almonds, stone fruit, and pome fruit), linamarin (cassava, lima beans, linseed/flaxseed, and spinach), linustatin (cassava, linseed/flaxseed), lotaustralin (cassava, lima beans), and prunasin (stone fruit, pome fruit, pip fruit). Additionally, cyanide is prevalent in sorghum, forage sorghum, Sudan grass, crosses, John's grass, and sweet sorghum [37]. While normally nontoxic, these cyanogenic glycosides can become toxic upon hydrolysis in livestock bodies [78, 79]. Factors such as rumen pH and microflora, rapid ingestion, consuming of immature cyanogenic plants, the amount of cyanogenic glycoside, the presence of glucosidase and free HCN, and the application of nitrogen fertilizers and herbicides can all contribute to increased cyanogenic toxicity in livestock [39, 77].

Almost all livestock, including cattle, sheep, and goats, are highly susceptible to poisoning from sorghum-type toxic plants. In contrast, horses and swine are generally not susceptible to such toxicity [37]. When livestock are poisoned, they experience anorexia, a condition characterized by a lack of oxygen in the issues, leading to eventual death. This occurs because cyanide binds with ferric ion (Fe+3) in cytochrome oxidase, inhibiting electron transport and oxygen transfer from oxyhemoglobin to tissues [38, 80]. Symptoms of cyanide toxicity in livestock include respiratory failure, restlessness, foaming at the mouth and nose, bright red blood, cerebral anoxia, muscle tremors, convulsions, inability to stand, and ultimately death [39]. Treatment for cyanide toxicosis involves administering intravenous sodium nitrate and sodium thiosulfate. To prevent and manage cyanide poisoning, it is crucial to implement good livestock management practices [37, 39].

#### 2.2.4. Nitrate and Nitrite Toxicity

Nitrate, a nitrogen-containing compound, can accumulate in plants depending on various factors. Soil nitrogen content, plant species, and maturity stage influence accumulation. Immature plants typically have higher nitrate concentrations than mature ones. Most nitrate is concentrated in the lower part of the plant, with leaves and flowers containing less. Herbicide use, wilting, and cloning can also affect nitrate levels [43]. Crops such as oat hay, sorghum, corn, and beets, as well as weeds like *P. purpureum* roots, pigweed (*Amaranthus* spp.), nightshades (*Solanum* spp.), *Chenopodium* spp., capeweed, lucerne, turnip, kochia, Russian thistle, and careless weed), are among the plants that contain nitrate [28, 39].

Nitrate and nitrite, compounds often found in plants, can cause toxicity in livestock particularly in buffalo, cattle, goats, and sheep. Horses and pigs are less susceptible due to their ability to convert nitrate to nitrite in the large intestine, reducing absorption into the bloodstream [81–84]. However, young livestock are at risk, especially after consuming nitrate-rich plants. In the rumen, microbes convert nitrite to ammonia. However, excessive nitrite can be absorbed into the bloodstream [43]. Nitrite combines with the ferrous ion (Fe+2) of hemoglobin, forming methemoglobin (ferric oxide iron (Fe+3) of hemoglobin), which prevents oxygen binding, leading to suffocation and death [85]. The methemoglobin complex avoids the binding of oxygen to hemoglobin, which makes deoxygenated blood (dark chocolate–colored blood) transported to tissues. Then, the poisoned livestock suffer from suffocation from the deprivation of oxygen and finally die from anoxia [39, 43, 86].

Livestock poisoned by nitrate and nitrite exhibit various clinical signs, including excessive salivation, bloating, difficulty breathing, staggering gait, dark brown blood, discolored mucous membranes, and congestion of the digestive organs. Ultimately, these livestock may suffocate and die. [28]. Treatment involves administering intravenously, which converts harmful methemoglobin back into functional hemoglobin [39].

#### 2.2.5. Photosensitization Effect

Photosensitization is a prevalent condition that affects the skin of livestock and reduces the quality of the skin and hides production. It is a type of severe skin inflammation caused by the sensitivity of skin cells and associated dermal tissues to sunlight after intake of toxic plants. The presence of photodynamic agents (chromophores) in the skin causes photosensitization [87, 88]. These chromophores absorb light energy and convert to a high-energy state and this energy generates reactive chemicals that sustain chemical processes in dermal components [72]. Livestock animals that lack fleece, hair, or pigments may be sensitive to toxic plants and develop lesions within minutes to hours after exposure. The skin, cornea, and mucous membrane of animals acquire photodynamic chemicals from toxic plants [89, 90].

Livestock can experience primary photosensitization after consuming toxic plants such as *Heracleum sphondylium, Hypericum erectum, Lotus corniculatus, Medicago sativa, Froelichia humboldtiana*, and *Biserrula pelecinus* [91]. When their nonpigmented skin is exposed to sunlight, photoactive chemicals in the plant react, causing skin damage (88). Secondary photosensitization occurs when protoporphyrin (phylloerythrin) accumulates in the skin. This is caused by the influence of toxic plants on the liver, leading to impaired bile excretion and damage to liver cells [90]. Common plants causing secondary photosensitization include *L. camara*, *Alternanthera philoxeroides*, *Brachiaria brizantha*, *B. decumbens*, *Brassica rapa, Enterolobium contortisiliquum*, and *Heliotropium europaeum* [91].

Livestock poisoned by toxic plants exhibit various symptoms including skin sloughing, severe itching, irritability, redness, swelling, fluid discharge, scarring, and skin necrosis [87, 92]. Treatment involves antibiotics for secondary infections, multivitamins, anti-inflammatory medications, and anthelmintic. Additionally, it is crucial to keep the affected animals away from toxic plants and injured animals and to provide shade to protect them from sunlight [43, 93].

#### 2.2.6. Effect on Urinary Tract

Toxic plants adversely effect on livestock's urinary tracts, leading to difficulties urinating, hematuria, and anuria. Plants containing cancer-causing and oxalate compounds are particularly harmful [15, 28]. These plants can cause urinary stones, which lead to intermittent blood in the urine, chronic bladder inflammation, painful urination, and potentially benign or malignant bladder tumors [5, 94].

##### 2.2.6.1. Bracken Ferns

They are found worldwide growing in burned regions, in woodlands, under the shadow of other plants, and on sandy soil ranges [39, 95]. Livestock, especially cattle and horses, are at risk of poisoning during summer when other food sources are scarce. The ferns contain toxic substances (thiaminase) that can degrade Vitamin B1 and cause blood loss, bone marrow damage, and ptaquiloside, and *cause* cancer in the urinary tract and digestive system. These cancerous tumors bleed, resulting in enzootic hematuria [50, 96]. Humans can also be affected by consuming the ferns or drinking milk from infected cows and by the fern's toxicity by eating its leaves, drinking milk from infected cows, or inhaling its spores [95, 97].

The symptoms of bracken fern poisoning vary depending on the animal species, making it a species-specific condition. In cattle, it can cause chronic blood in the urine and bladder tumors. Sheep and cattle can experience acute hemorrhagic illness and upper digestive tract cancer. Additionally, sheep and horses may suffer from thiamine deficiency and kidney atrophy [50, 98]. Beyond urinary tract issues, bracken ferns can cause incoordination, lethargy, and a crouched posture in horses, leading to muscle and nerve problems.

Muscle tremors can develop, and severe cases can be fatal within weeks [99]. Treatment for bracken fern poisoning involves supportive care, such as saline laxatives, activated charcoal, and medications to stop bleeding and support blood health (copper, cobalt, and iron). Antibiotics are also used to prevent secondary infections. Additionally, intravenous thiamine injections can help counteract the vitamin deficiency caused by the ferns [50, 100].

##### 2.2.6.2. Oxalate Plant

Oxalate compounds are found in the plants in the form of oxalic acid. There are two types of oxalic acids: soluble and insoluble. Soluble oxalate binds with monovalent elements such as potassium, sodium, and ammonium, while insoluble oxalate binds with divalent elements such as iron, calcium, and magnesium [101]. High levels of soluble oxalate accumulate in toxic plants belonging to the Poaceae, Amaranthaceae, and Polygonaceae families. The leaves of certain acacia and grass species, commonly cultivated in tropical and subtropical areas, contain the highest amount of soluble oxalate [102, 103]. Some common oxalate plants that cause toxicity in livestock ruminants include *Amaranthus*, *Steria sphacelate*, *P. clandestinum*, *Panicum humidicola*, *Rumex* spp., *Cenchrus ciliaris*, *Brachia humidicola*, *Digitaria decumbens*, *Curyl*, Bassia (*Bassia hyssopifolia*), and *Sarcobatus vermiculatus* [5, 44].

Ruminants are generally less susceptible to oxalate toxicity compared to nonruminant animals. This is due to their rumen containing microflora that can be convert oxalate into harmless substances such as formic acid and carbon diodide. Nonruminant lack this ability, making them more susceptible to toxicity.However, factors such as the amount of oxalate-rich plants consumed, the concentration of oxalate in these plants, and the health of the ruminant can influence their susceptibility to oxalate toxicity. Young animals are more vulnerable to oxalate poisoning compared to adults [104]. Two bacteria species, *Oxalobacter formigenes* and *Enterococcus faecalis*, play a significant role in oxalate degradation within the rumen and intestine of ruminants. However, if ruminants consume excessive amounts of oxalate-rich plants, the rumen microorganisms may be overwhelmed, leading to oxalate accumulation in the bloodstream. This can result in various health issues, including hypocalcemia and kidney damage [104–106].

The most common symptoms of oxalate poisoning that affect the urinary system include anuria, uremia, hypocalcemia, hypomagnesaemia (grass tetany), urolithiasis in males, acute renal failure, weakness, ataxia, high blood urea levels, proteinuria, perineal edema, and nephrosis [1, 104]. Good management practices can help prevent oxalate poisoning in livestock. When animals are fed low-oxalate forage, they should only eat and drink. When they are hungry or thirsty, rather than when they are eating oxalate-rich plants. To avoid the formation of urinary calculi, drinking enough water is recommended [107].

### 2.3. Managements of Toxic Plants

#### 2.3.1. Treatment

Livestock poisoned by toxic plants are traditionally treated by healers using therapeutic plants, soap, burned plant leaves (ash), and drenching charcoal [108]. In most cases of plant poisoning, treatment focuses on symptomatic and supportive care, as most toxins lack specific antidotes. Due to high costs and limited availability, veterinary practitioners often cannot obtain clinically viable antidotes [28, 39]. Livestock poisoned by lethal plant toxins require immediate treatment to prevent death. Supportive therapy and available antidotes may be used to manage toxicity, as indicated in Table 2.

#### 2.3.2. Management Approach to Toxic Plants

Managing toxic plants in livestock health requires a comprehensive approach emphasizing prevention, early detection, and appropriate treatment. The primary strategy for controlling the effects of toxic plants on livestock involves various measures, including the destruction of toxic plants and preventing livestock access to toxic plants. These measures can be used to identify toxic plants on rangelands, assess their condition and season of occurrence, improve rangelands with grazing plants to prevent poisoning, graze the rangelands at appropriate times without overgrazing, prevent stressed and burdened livestock from grazing in toxic plant-infested areas, and provide salts and other supplements for the prevention and control of toxic plants in livestock health management [32, 109]. Toxic plants can be controlled through proactive and multifaceted approaches. Key strategies in the management of toxic plants include the following [23, 28, 110–112].

##### 2.3.2.1. Toxic Plant Monitoring and Management

Regularly identify and inspect pastures, rangelands, and feed sources for the presence of toxic plants. Track changes in their size and distribution throughout the season to understand the factors influencing their growth and spread. This information can help prevent the establishment and spread of new infestations.

##### 2.3.2.2. Grazing and Pasture Management

Rotational grazing system is used to limit animal access to the risk of toxic plants. This prevents overgrazing and encourages the growth of desirable forage species that can outcompete toxic plants. Pasture improvement promotes the growth of nutritious forages. This can be achieved through soil testing, fertilization, reseeding with beneficial plants, and irrigation. This enhances animal nutrition and reduces the likelihood of livestock seeking out toxic plants. Seed bank management adopts strategies to prevent the buildup of toxic plant seeds in the soil, such as managing pastures before flowering. This can significantly reduce the persistence of toxic plants over time. Detect and remove toxic plants before they produce seeds to limit the number of viable seeds entering the soil.

##### 2.3.2.3. Physical and Chemical Control

Toxic plants can be eradicated through a combination of physical and chemical methods. Manual removal is effective, but it is essential to eliminate the entire plant, including root systems. Alternatively, machinery and herbicides can be employed for eradication. While herbicides can be used, it is crucial to adhere to guidelines and regulations. Contact herbicides offer specific plants, whereas systemic herbicides are absorbed by plants and provide longer-term control.

##### 2.3.2.4. Feed Management and Giving Awareness

Feed management plays a crucial role in preventing toxic plant contamination. This involves inspecting stored feed and forages for the presence of toxic plants. Proper storage and handling practices are essential to safeguard feed from contamination. Additionally, raising awareness among livestock owners, farmers, and ranchers about toxic plant identification, their associated risks, and appropriate control strategies is imperative.

### 2.4. Economic Importance of Toxic Plants

The economic impact of poisonous plants on livestock production can fluctuate based on various factors, including geographical location, the specific plant species, the severity of toxicity, and the scale of the livestock operations. Toxic plants have a significant negative impact on livestock production and their derived products. These plants inflict both direct and indirect economic losses [8, 57].

#### 2.4.1. Direct Economic Losses

The mortality of livestock and diminished productivity resulting from the ingestion of poisonous plants can lead to immediate financial losses. These losses may encompass the cost of deceased animals, reduced weight gain, decreased milk production, lower fertility rates, and reproductive impairments. Precisely estimating these direct losses can be challenging due to the influence of factors such as the number of affected animals, the toxicity level of the plant, and market prices for livestock products [57, 69].

Toxic plants pose a significant threat to livestock health and the agricultural economy, causing annual losses of around 250 million dollars due to deaths, miscarriages, and decreased productivity. High neonatal mortality and congenital malformations are major factors driving livestock population decline [23, 113]. Additionally, toxic plants damage hides and wool, reducing their market value and negatively impacting the economy. Sheep wool production and livestock productivity also suffer, as do the quality and value of the liver and other organs, leading to weight loss in cattle [114]. Toxic plants also contaminate milk, leading to decreased quality and market acceptance, potentially harming both children and adults [57, 115–117]. Several toxic plants contribute to this issue, with common toxins excreted in milk including tremetol, glucosinolates, piperidine alkaloids, nicotianas, and pyrrolizidine alkaloids. Tremetol, found in white snakeroot and rayless goldenrod (Table 3), is particularly harmful, causing human poisoning and affecting cows and their calves [121].

#### 2.4.2. Indirect Losses

The consumption of toxic plants by livestock can lead to indirect economic losses due to increased costs associated with veterinary services, additional feed requirements, diagnostic testing, treatment expenses, and labor involved in managing affected animals. Additionally, toxic plant ingestion can result in secondary health problems, such as liver damage and weakened immune function, further impact livestock productivity, and increase treatment costs [8, 114]. The most significant economic burden often arises from managing toxic plants, which may involve using herbicides, preventing toxicity by decreasing pasture productivity through spraying, reducing farm carrying capacity by closing off grazing areas, and providing alternative feed [4].

### 2.5. Status of Toxic Plants in Ethiopia

Numerous studies have aimed to identify medicinal plants with potential antimicrobial properties. However, the toxicity of these plants often receives less attention than their in vitro screening for antimicrobial activity. Consequently, some medicinal plants may pose risks to livestock through various mechanisms [16, 123–126]. The severity of these effects can vary depending on factors including plant species, developmental stage, and environmental conditions. Ethiopia has a diverse plant life, some of which can be harmful to livestock when ingested in large quantities or under specific circumstances. Research has mapped the distribution and prevalence of these toxic plants, which can have severe consequences for the nation's livestock population [13, 123, 124]. Grazing animals in grasslands and forests are particularly susceptible to exposure to these harmful plants. Toxic plants are widespread across Ethiopia, particularly in the country's diverse forests. Decades of research have consistently shown that these plants have a significant negative impact on livestock health and the nation's economy [12, 13]. Recent research has investigated the distribution and impact of toxic plants on livestock health in Ethiopia. Findings indicate that the most prevalent species of toxic plants are found in the western, eastern, northern, and southern regions of the country. The most common toxic plants that were assessed in different districts of Ethiopia are indicated in Table 4.

## 3. Conclusion and Recommendations

Plants have long been recognized for their potential to treat livestock diseases. However, the misuse of medicinal plants and exposure to toxic plants pose significant risks to livestock health. The toxicity of plants can vary widely depending on factors such as the season, geographical location, plant species, dosage, and frequency of exposure. These toxins can disrupt various physiological systems, including the digestive, hepatic, integumentary, urinary, circulatory, reproductive, and respiratory systems. While supportive therapy and antidotes can be helpful in some cases, many toxic plants lack effective antidotes, making prevention and management crucial. Therefore, it is imperative to assess the distribution of toxic plants and implement strategies to control and prevention their impact on livestock health and productivity. This includes promoting awareness among livestock owners, communities, and government agencies, as well as supporting research and development of effective management techniques. Addressing these issues can minimize economic losses from toxic plant-related livestock diseases and safeguard animals' and humans' well-being.

## Figures and Tables

**Table 1:** The common toxic plants and their secondary metabolites.

Species	Toxic compound	Susceptible animals	Clinical signs	Reference
*Crotalaria*	Pyrrolidine alkaloids	All livestock	Hepatitis, a hypotonic tongue, circling, frequent yawning	[36]
*Cyanogenic plants, Prunus africanus, Sudan grass*	Amygdalin, prunacin, linamarin, dhurrin, and taxiphyllin	All livestock	Labored breathing, dyspnea, restlessness, tremors, terminal clonic convulsions, and opisthotonus	[37, 38]
*Datura stramonium*	Tropane alkaloids	Cattle, horses	Flushed skin, dilated pupils, respiratory failure, death	[39]
*Lantana camara*	Pentacyclic triterpenes	All livestock	Jaundice and photosensitization	[40]
*Locoweeds*	Swainsonine and indolizidine alkaloids	Cattle	Placental resorption, abortion, and feral birth defect	[41]
*Lupinus* spp.	Quinolizidine alkaloids	Sheep, cattle	Teratogenic effects	[42]
*Nitrate containing plants*	Nitrate, later converted to nitrite by bacteria	Cattle, sheep	Dyspnea, salivation, tremors, staggering, bloat	[28, 43],
*Oxalate-*containing plants*, Pennisetum* spp.*, Cenchrus ciliaris, Digitaria decumbens, Amaranthus hybridus*	Oxalate crystal	All livestock	Weakness, ataxia, anuria, uremia, proteinuria, perineal edema, and renal failures	[44, 45]
*Parthenium hysterophorus*	Germacrene D, *trans*-b-ocimene, b-myrcene	Cattle, horses	Dermatitis, inflammation, eczema, asthma, hay fever	[46, 47]
*Poison hemlock*	Piperidine alkaloids	Cattle, sheep	Ataxia, frequent urination and defecation, salivation, death, abortion, birth defects	[41, 48]
*Prunus africana*	Phytosterols, tannins	Cattle, goat	Gastrointestinal upset, potential for liver damage	[49]
*Pteridium aquilinum*	Ptaquiloside	Cattle, sheep	Hemorrhagic syndrome, cancer risk in chronic exposure	[50]
*Ranunculus sardous*	Protoanemonin	Cattle, sheep	Oral irritation, vomiting, diarrhea, dermatitis, death	[51]
*Ricinus communis*	Ricin, cardiac glycoside	All livestock	Nausea, muscle spasms, purgation, convulsions, death	[52, 53]
*Solanum nigrum*	Solanine, glycoalkaloids	All livestock	Gastrointestinal symptoms, neurological effects	[54]
*Veratrum*	Cyclopamine and steroidal alkaloids	Sheep and goat	Birth defects, abortion	[28]
*Xanthium strumarium*	Hydroquinone in seeds and seedlings	All livestock	DNA damage, chromosome aberrations	[46, 55]

**Table 2:** The commonly used antidote and supportive therapy for poisoned livestock.

Toxic compound	Toxicity effects	Treatment	References
Cyanogenic glycosides	Hematotoxic	Sodium nitrate and sodium thiosulfate, activated charcoal	[37]
Tropane alkaloids	Anticholinergic	Physostigmine, activated charcoal	[39]
Lantadene A and B	Hepatotoxicity	Liver supplements, fluids, and electrolytes	[40]
Nitrates and nitrites	Hematotoxic	Methylene blue, activated charcoal	[43]
Ricin, ricinine	Gastrointestinal toxicity	Symptomatic and supportive care, activated charcoal	[43, 53]
Thiaminase inhibitors and ptaquiloside	Aplastic anemia., bovine enzootic hematuria, bladder carcinoma	Thiamine, batyl alcohol	[100]

**Table 3:** The common plant toxicant excreted in milk.

Name of toxins	Species of toxic plant	References
Glucosinolates	*Brassica* spp.	[118]
	*Armoracia* spp.	

Tremetol	*Eupatorium* spp.	[119, 120]
	*Isocoma* spp.	
Piperidine alkaloids	*Conium maculatum*	
	*Nicotianas* spp.	

Pyrrolizidine alkaloids	*Crotalaria* spp.	[65]
	*Heliotropium europaeum*	
	*Festuca* spp.	
	*Cynoglossum* spp.	
	*Symphytum* spp.	
	*Echium* spp.	
	*Senecio* spp.	

Quinolizidine alkaloids	*Astragalus*	[121, 122]
	*Oonopsis*	
	*Stanleya*	
	*Muchaeranthera*	

Sesquiterpene lactones	Rubber weed	[116]
	Bitter weed	

**Table 4:** The most common toxic plants reported in Ethiopia.

Scientific name	Poisonous parts	Susceptible livestock	Poisoning effect	Study area	Reference
*Arisaema ennaephyllum*	Seed, fruit	Cattle, goat	Respiratory failure, coma, death	H/Guduru Wollega, Oromia region	[127]
*Datura stramonium*	Seed, fruit	Cattle	Pupils dilate, thirst, dry and burning skin, bloating, dyspnea	Kellem Wollega, Oromia region	[128]
*Prunus african*	Leaf, bark	Cattle, sheep, goat	Salivation, bloating, colic, diarrhea, loss of appetite	H/Guduru Wollega, Oromia region	[127, 129]
*Ranunculus sardous*	Leaf	Cattle, sheep	Oral and gastro intestinal irritation, bloating, salivation, colic	Kellem Wollega, Oromia region	[128]
*Ricinus communis*	Seed, fruit	Cattle, sheep, goat, equine	Salivation, diarrhea, mydriasis, teeth grinding, abdominal pain, sweating, death	H/Guduru, Kellem Wollega, Oromia region	[127, 128]
*Pteridium aquilinum*	Leaf	Cattle, horse, donkey, sheep	Anemia, blood urine, salivation	Woliso, Southwest Shewa; H/Guduru, Kellem Wollega, Oromia region	[127, 128, 130]
*Conium maculatum*	Leaf	All	Diarrhea, ataxia, salivation, coma		
*Xanthium strumarium*	Seed and leaf	Cattle, sheep, goat	Vomiting, diarrhea, respiratory distress, trebling, coma, death	Wondo Genet, Sidama region	[25]
*Parthenium hysterophorus*	Leaf	All	Anorexia, allergy, salivation, sour milk	Woliso, Southwest Shewa; Kellem Wollega, Oromia region	[128, 130]
*Sorghum bicolar*	Leaf	Cattle, sheep	Bloating, dyspnea		
*Lantana camara*	All parts	Cattle	Photosensitization	Kellem Wollega, Oromia region	[128]
*Hibiscus esculentus*	Leaf	All	Bloating	Woliso, Southwest Shewa, Oromia region	[130]
*Argemone mexicana*	All parts	Cattle, sheep, goat	Weakness, hemorrhagic enteritis, death	Wondo Genet, Sidama region	[25]
*Euphorbia tirucalli*	All parts	Cattle, sheep, goat	Abortion, skin loss, blindness		
*Stephania abyssinica*	All parts	Sheep	Vomiting, nausea		
*Xanthium spinosum*	All parts	Horse, donkey	Restless, death	H/Guduru Wollega, Oromia region	[129]
*Allium cepa*	Root	Cattle, sheep, goat	Diarrhea, inappetance		
*Allophylus apsinicu*	Leaf	All	Ataxia, death		[127, 129]
*Crotalaria incana*	Seed, fruit	All	Depression, tachycardia, and diarrhea	H/Guduru Wollega, in and around Adama East Shewa, Oromia region	[49, 129]
*Ponderosa pine*	Leaf	Cattle	Abortion	Kellem Wollega, Oromia region	[128]
*Rumex abyssinicus*	Whole	Cattle	Weakness, dullness, incoordination	Woliso, Southwest Shewa, Oromia region	[130]
*Salix subserrata*	Leaf	Cattle	Bloody urine		
*Loudetia flavida*	All part	All	Urinate blood	Woliso, Southwest Shewa; Borana, Oromia region	[130, 131]
*Capparis tomentosa*	Leaf, seed pod	Camel	Bloating, death	Afar region	[132]
*Maytenus senegalensis*	Leaf	All	Blood urine		

## Data Availability

This is a review manuscript, and the data in this manuscript were taken from different journals with proper acknowledgment of the authors.

## Nomenclature

ATP: Adenosine triphosphate
BC: Before Christ
BEH: Enzootic hematuria
CYP: Cytochrome P450
DNA: Deoxyribose nucleic acid
GIT: Gastrointestine tract
HCN: Hydrocyanic acid
PAs: Pyrrolizidine alkaloids
pH: Power of hydrogen
RNA: Ribonucleic acid
