# Corrigendum to “Toxic Plants and Their Impact on Livestock Health and Economic Losses: A Comprehensive Review”

**DOI:** 10.1155/jt/9787082

**Published:** 2025-07-24

T. Abdisa and T. Dilbato Dinbiso, “Toxic Plants and Their Impact on Livestock Health and Economic Losses: A Comprehensive Review,” *Journal of Toxicology* 2024, no. 1 (2024): 1–15, https://doi.org/10.1155/jt/9857933.

In the article titled “Toxic Plants and Their Impact on Livestock Health and Economic Losses: A Comprehensive Review,” author Tegegn Dilbato Dinbiso was affiliated to Ambo University, Guder Mamo Mezemir Campus, Department of Veterinary Science, West Shewa Zone, Oromia, Ethiopia, which is incorrect. The correct affiliation for this author is as follows:

Ambo University, Guder Mamo Mezemir Campus, School of Veterinary Medicine, Department of Veterinary Science, Ambo, Oromia, Ethiopia

We apologize for this error.

